# The Influence of Structural Variants of the *CES1* Gene on the Pharmacokinetics of Enalapril, Presumably Due to Linkage Disequilibrium with the Intronic rs2244613

**DOI:** 10.3390/genes13122225

**Published:** 2022-11-27

**Authors:** Anna Ikonnikova, Ruslan Kazakov, Tatiana Rodina, Artem Dmitriev, Evgeniy Melnikov, Alexander Zasedatelev, Tatiana Nasedkina

**Affiliations:** 1 Engelhardt Institute of Molecular Biology, Russian Academy of Sciences, 119991 Moscow, Russia; 2 Federal State Budgetary Institution “Scientific Centre for Expert Evaluation of Medicinal Products” of the Ministry of Health of the Russian Federation, 127051 Moscow, Russia; 3 Institute of Pharmacy of I. M. Sechenov First MSMU of the Ministry of Health of the Russian Federation (Sechenov University), 119435 Moscow, Russia

**Keywords:** carboxylesterase 1, *CES1*, *CES1P1*, *CES1A2*, rs2244613, enalapril, enalaprilat, SNP, pharmacogenetics, cardiology

## Abstract

Variants in the *CES1* gene encoding carboxylesterase 1 may affect the metabolism of enalapril to the active metabolite enalaprilat. It was shown that the A allele of rs71647871 and the C allele of rs2244613 led to a decrease in plasma enalaprilat concentrations. This study aimed to estimate the effect of structural haplotypes of *CES1* containing the pseudogene *CES1P1,* or a hybrid of the gene and the pseudogene *CES1A2,* on the pharmacokinetics of enalapril. We included 286 Caucasian patients with arterial hypertension treated with enalapril. Genotyping was performed using real-time PCR and long-range PCR. Peak and trough plasma enalaprilat concentrations were lower in carriers of *CES1A2*. The studied haplotypes were in linkage disequilibrium with rs2244613: generally, the A allele was in the haplotype containing the *CES1P1*, and the C allele was in the haplotype with the *CES1A2*. Thus, carriers of *CES1A2* have reduced CES1 activity against enalapril. Linkage disequilibrium of the haplotype containing the *CES1P1* or *CES1A2* with rs2244613 should be taken into account when genotyping the *CES1* gene.

## 1. Introduction

Carboxylesterase 1 (CES1) is a serine esterase that controls most of the hydrolytic activity in the human liver. It is involved in many important functions, such as drug metabolism and the metabolism of other xenobiotics, as well as endogenous biomolecules [1].

It has been shown that the activity of the enzyme can critically depend on the genetic variants of the *CES1* gene. Accordingly, markers in the *CES1* gene play a role in interindividual differences in the metabolism and effect numerous drugs that are substrates of this enzyme [2,3,4,5,6,7,8,9,10,11,12,13,14,15,16,17,18,19,20,21]. Thus, single nucleotide polymorphisms (SNPs) are known in the *CES1* gene that lead to a change in the activity of the enzyme, for example, G143E (rs71647871) [2,3,4,5,6,7,8,9,10,11] and variant 1168-33C>A in intron 10 (rs2244613) [12,13,14,15,16,17,18,20].

**Figure 1:**
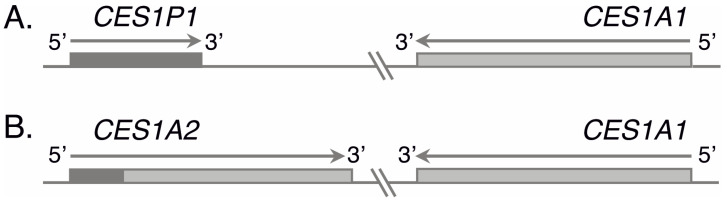
Main structural haplotypes of *CES1* containing *CES1P1* (**A**) or *CES1A2* (**B**). Light gray and dark gray show regions corresponding to the *CES1A1* and *CES1P1* sequence, respectively.

The chromosomal region containing the *CES1* gene has a complex structure (Figure 1). In general, there are two main haplotypes: in one of them, the *CES1* gene (so-called *CES1A1*) is located tail-to-tail with a nonfunctional pseudogene (*CES1P1*); and in the other, it is located with a hybrid of the gene and the pseudogene (*CES1A2*, also called *CES1P1VAR*) [22,23,24]. Most of the *CES1A2* coding sequence is identical to *CES1A1*, while the promoter region, exon 1, and the first part of intron 1 are homologous to *CES1P1*. *CES1A2* has a low transcriptional activity (2% of the *CES1* gene activity) [25], and although there have been reports of a variant with increased promoter activity (due to SP1 binding sites of transcription factors) [26], the experimental evidence for its real functional value and potential clinical utility is limited. However, in some studies, *CES1A2* was considered as a duplication of the *CES1* gene or a functional copy of the gene [8,27].

Studies on how the presence of *CES1A2* affects the metabolism of CES1 substrates have shown inconsistent results: no effect on enalapril [3,27] or oseltamivir [28], increased enzymatic activity for irinotecan [19], and even decreased activity for methylphenidate in subjects with homozygous duplication of *CES1* [8]. The latter unexpected observation may be due to the possible influence of undiscovered genetic variants affecting the activity of the enzyme. Thus, the association of *CES1* structural variants with interindividual variability in enzyme function remains controversial.

With regard to the pharmacogenetics of enalapril, *CES1* genetic variants may influence the rate of hydrolysis of enalapril, in relation to the active metabolite enalaprilat. Thus, it was found that rs71647871 and rs2244613 affect the plasma concentrations of enalaprilat [2,3,4,12]. Two studies analyzed the number of copies of the *CES1* gene, and there were no associations with the pharmacokinetics of enalapril [3,27]. However, it is worth noting that these studies were conducted on liver samples [3] and healthy volunteers [27]. We previously studied the effect of three genetic markers in the *CES1* gene (rs2244613 and rs71647871 and the variant with a weak promoter, *CES1A1c*) on the pharmacokinetics of enalapril in patients with arterial hypertension [12]. A decrease in peak and trough enalaprilat concentrations in carriers of the C allele rs2244613 was found, as well as a decrease in enalaprilat trough concentrations in *CES1A1c* homozygotes. In this work, our goal was to supplement the obtained knowledge by studying the effect of structural haplotypes of *CES1* containing *CES1P1* or *CES1A2* on the pharmacokinetics of enalapril.

## 2. Materials and Methods

### 2.1. Patients and Pharmacokinetic Study

The patient cohort, pharmacokinetic study, and DNA extraction were described in our previous work [12]. Briefly, we studied 286 Caucasian patients with grades 1–3 of arterial hypertension, who received enalapril (2.5 to 20 mg twice a day) and underwent therapeutic drug monitoring on day 3, while taking enalapril. The plasma concentration of enalapril and enalaprilat was determined before the following intake of the drug and 4 h after it (trough and peak concentrations of enalaprilat, respectively). The analysis used a triple quadrupole liquid chromatograph–mass spectrometer, Nexera LCMS-8040 (QQQ) (Shimadzu, Kyoto, Japan). Genomic DNA was extracted from blood collected in EDTA-containing tubes using the QIamp DNA Mini kit (Qiagen, Hilden, Germany) or LumiPure genomic DNA Blood and Buccal Kit (Lumiprobe RUS Ltd., Moscow, Russia).

This study was approved by the Institutional Review Board of the Federal State Budgetary Institution “Scientific Centre for Expert Evaluation of Medicinal Products” of the Ministry of Health of the Russian Federation (protocol 2018/04 and date of approval 17 January 2018). All participants provided written informed consent. The study was performed in accordance with the World Medical Association Declaration of Helsinki.

### 2.2. Genotyping

The copy number of *CES1P1* was determined by quantitative real-time PCR on a LightCycler^®^ 96 (Roche, Basel, Switzerland). We used primers and probes to the sixth exon of *CES1P1* and normalized to the *F8* gene [29] located on the X chromosome. The PCR mixture (25 μL) contained 1×PCR buffer (Syntol, Moscow, Russia), 3.25 mM MgCl2, 0.4 mM of each dNTP (New England Biolabs, Hitchin, UK), 2.5 units of SynTaq DNA polymerase (Syntol), 5 pmol of each primer, 4 pmol of each TaqMan probe, and 1 μL of DNA (30 ng/mL). Primer and probe sequences were as follows: forward primer 5′- TGA GTT CTG TGG ACC CAC TTG T-3′, reverse primer 5′-CCC TGG TTA TCA GGC GCT C-3′, and TaqMan probe 5′- FAM-TGT GGG CCT GAA GCA GTT CCA CA-BHQ1-3′ for *CES1P1*; forward primer 5′- CTA CCA TCC AGG CTG AGG TTT ATG -3′, reverse primer 5′- CAC CAA CAG CAT GAA GAC TGA CA -3′, and TaqMan probe 5′- ROX-ACA GTG GTC ATT ACA CTT AAG AAC ATG GCT TCC C-BHQ2-3′ for *F8*. All primers and probes were synthesized and purified by DNA-Synthesis (Russia). The PCR program was as follows: preincubation 95 °C for 3 min, then 45 cycles of 95 °C for 15 s, and 60 °C for 20 s. Detection was carried out on channels FAM and Red610. Determination of copy number *CES1P1* was carried out using LightCycler^®^ 96 Roche software (version 1.1.0.1320).

We inferred the number of *CES1A2* copies based on the number of *CES1P1*, since it is known that in a diplotype, there can be either two *CES1P1*, or one *CES1P1* and one *CES1A2*, or two *CES1A2*. Additionally, we confirmed the presence or absence of *CES1A2* by long-range PCR for a 2.6 kb fragment from the promoter to intron 1 of *CES1A2,* using primers from Bjerre et al. [30]: forward 5′- CAG GAG CTA TTG AGA GAT GGA A×T×C× A×T-3′ (× – is a phosphorothioate bond) and reverse 5′- GGA CTG TGA GGG TAC ATA CGG-3′. The PCR mixture (15 μL) contained 1×PCR buffer (New England Biolabs), 0.5 mM of each dNTP (New England Biolabs), 2.5 units of LongAmp^®^ Taq DNA Polymerase (New England Biolabs), 6 pmol of each primer, and 1 μL of DNA (30 ng/mL). The PCR program was as follows: preincubation 93 °C for 3 min, then 10 cycles of 93 °C for 15 s, 57 °C for 30 s, and 68 °C for 3 min, then 20 cycles of 93 °C for 15 s, 57 °C for 30 s, and 68 °C for 4 min. The presence of the product was checked by electrophoresis in 1% agarose gel.

### 2.3. Statistical Analysis

Multivariable linear regression analysis was used to assess the effect of the number of *CES1A2* copies on peak and trough enalaprilat concentrations. The pharmacokinetic parameters (enalaprilat concentrations) were logarithmically transformed before analysis. Comparison of linear regression models was carried out using ANOVA. Statistical analysis was performed in R (version 4.1.1; R Foundation for Statistical Computing, Vienna, Austria) using the package “genetics” and in JASP (Version 0.16.3) computer software. Differences were considered statistically significant when *p* was below 0.05.

## 3. Results

In total, 286 samples were genotyped. Distribution of diplotypes corresponded to the Hardy–Weinberg equilibrium (Table 1).

Comparing these results with our previous work [12], we found that the haplotype containing *CES1P1* or *CES1A2* was in linkage disequilibrium with rs2244613 (R^2^ = 0.779 and D’= 0.988). The A allele rs2244613 was in the haplotype containing the pseudogene *CES1P1*, and the C allele was in the haplotype with the *CES1A2* in 96% of cases (Table 2). Only in 4% of cases was it the other way around. Linkage disequilibrium analysis of all studied genetic markers (rs2244613, rs71647871, *CES1A1c,* and the haplotype containing *CES1P1* or *CES1A2*) is shown in Figure S1.

Since we had previously shown a decrease in enalaprilat plasma concentrations in the same cohort of patients in carriers of the C allele rs2244613 in a linear regression analysis [12], in this study, we used the same covariates for linear regression models, but instead of the rs2244613 genotype, we used the *CES1A2* copy number. For peak concentration, in addition to *CES1A2*, the model included age, sex, single dose, and the presence of the *CES1A1c* variant (Table 3); for the trough concentration, it included age, single dose, and *CES1A1c* (Table 4). It can be seen that the presence of *CES1A2* was also associated with a decrease in plasma concentrations of enalaprilat: for peak concentration, in carriers of one or two copies of *CES1A2* (Table 3); for trough concentration, a statistically significant decrease was found in carriers of two copies (Table 4). As enalaprilat concentrations were logarithmically transformed, coefficients for genotypes can be interpreted as a percentage change in concentration (when the coefficient is multiplied by 100).

Next, we compared the influence of *CES1A2* and rs2244613 as predictors, by adding them to the initial linear regression models, including all other predictors of the respective models for peak and trough enalaprilat concentrations. As can be seen from Table 5, adding both predictors led to a statistically significant increase in R^2^, and their effects were comparable.

Table 3 and Table 4 also show decreased enalaprilat peak and trough concentrations in *CES1A1c* homozygotes, whereas when using rs2244613 as a predictor, the effect of this genotype on peak concentration was statistically significant, but for the trough concentration it was “at the level of the trend towards significance” [12].

In a linear regression analysis of enalapril trough concentrations, we did not find a statistically significant effect for any of the studied genetic markers (rs2244613, rs71647871, *CES1A1c,* and number of copies of *CES1A2*). In our study, therapeutic drug monitoring was not carried out at a time point corresponding to the peak concentration of enalapril, so analysis of this parameter was not performed.

## 4. Discussion

In the current work, we aimed to evaluate the effect of structural haplotypes of *CES1* containing *CES1P1* or *CES1A2* on the pharmacokinetics of enalapril in patients with arterial hypertension. We found that, in Caucasians, the presence of *CES1P1* or *CES1A2* in the haplotype was in linkage disequilibrium with rs2244613. Generally, the A allele rs2244613 was in the haplotype containing the pseudogene *CES1P1*, and the C allele was in the haplotype with the *CES1A2* (Table 2). We have previously shown that rs2244613 is significantly associated with plasma enalaprilat concentrations: peak and trough enalaprilat concentrations were reduced in carriers of the C allele rs2244613 [12]. In this work, we studied the same cohort of patients. Accordingly, here, we show that enalaprilat concentrations were reduced in *CES1A2* carriers. When considering *CES1A2* as a functional copy of the gene, this result may seem contradictory. However, Stage et al. [8] also found that the median AUC of d-methylphenidate was significantly larger in a group with four *CES1* copies (*CES1A2/CES1A2* diplotype) than in that with two or three copies, suggesting a significantly decreased enzyme activity. The authors suggested that this may be a reflection of an undiscovered mutation affecting the activity of the enzyme. According to our results, rs2244613 may be such a genetic variant. Since a genome-wide association study has shown its effect on the pharmacokinetics and drug response of dabigatran [13], many studies have confirmed its effect on CES1 activity, not only for dabigatran [15,16,17,20] but also for other drugs [12,14,18]. However, intronic variant rs2244613 cannot definitely explain the mechanism for the decrease in enzyme activity. It is possible that this marker may affect post-transcriptional processes such as splicing or there may be a linkage disequilibrium with another variant that is causal.

Our observations about linkage disequilibrium raise questions regarding approaches to *CES1* genotyping. Due to the high homology with the *CES1A1* gene, *CES1A2* can cause difficulties in genotyping, and many genotyping procedures appear to lack specificity [23]. A special genotyping approach that takes structural variation into consideration has been developed by researchers from the INDICES Consortium to solve this problem [24,30]. This procedure involves determining the copy number of the *CES1* gene (i.e., *CES1A1*+*CES1A2*). Next, a 12.5 kb fragment from promoter to intron 5 of *CES1A1* gene is amplified for all samples using the long-range PCR method. The 19.2 kb *CES1A1* fragment from intron 5 to 3′ UTR is only amplified for samples without *CES1A2* (i.e., with two copies of *CES1P1*), since there is complete homology between *CES1A1* and *CES1A2* in this region. Furthermore, the obtained fragments are used for further analysis via Sanger sequencing. This procedure allows a highly specific analysis of the *CES1A1* gene. However, there is a problem, where by SNPs in the region from intron 5 to 3′ UTR in samples that contain *CES1A2* are not analyzed. As we have shown with the example of rs2244613 that haplotypes containing *CES1P1* or *CES1A2* can be in linkage disequilibrium with other SNPs, the exclusion of samples with *CES1A2* from an analysis can lead to distortion of genotyping results, since information about SNPs in the region from intron 5 to 3′ UTR is lost.

Thus, in studies using this genotyping approach, the authors noticed that, for rs2244613, a significantly lower frequency of allele C was found than in other studies [9,22,31]. We assume that this is probably due to the exclusion of samples with *CES1A2* from the analysis of this gene region, because the C allele is predominantly found in the haplotype containing *CES1A2*. The C allele frequency of 3.5–4.9% in these studies [9,22,31] is consistent with the frequency of haplotype containing *CES1P1* and the C allele rs2244613 in our study (3.85%) (Table 2), whereas studies of Caucasians (or predominantly Caucasians), without exclusion of samples with *CES1A2*, showed a frequency of the C allele of about 20% [12,13,16,17,18,21].

**Figure 2:**
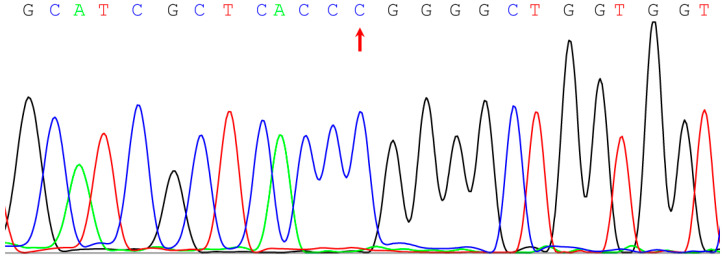
Sanger sequencing chromatogram for a sample with two copies of *CES1A2*. The arrow shows rs2244613.

It is worth noting that Sanger sequencing of samples with two copies of *CES1A2* and the CC rs2244613 genotype did not show the presence of any peaks other than C (Figure 2); that is, this nucleotide was located both in intron 10 of *CES1A1* and in the corresponding region of *CES1A2*. However, in our cohort, there was one patient with two copies of *CES1A2*, but with the AC rs2244613 genotype. In this case, we could not determine the regions in which A and C were located. For further study of the structure of the *CES1* gene, it would be advisable to use long-read sequencing technologies [32]. This is a promising method in the field of pharmacogenomics research, although it has mainly been used to analyze the *CYP2D6* gene.

Our study has some limitations. We included only individual SNPs in the study in the analysis, while sequencing could have provided more meaningful information. However, Sanger sequencing and high-throughput short-read sequencing cannot determine whether SNPs are located in *CES1A1* or *CES1A2* (for regions from intron 5 to 3′-UTR in *CES1A2* carriers), due to the high homology of these regions. Therefore, we believe that, in the future, it will be necessary to use long-read sequencing. We would also like to note that we showed linkage disequilibrium of *CES1A2* and rs2244613 in Caucasian patients, and this may differ in other ethnic groups. In East Asia, in contrast to Europe, the A rs2244613 allele is minor (frequency is about 40%) [15].

## 5. Conclusions

We showed that the presence of *CES1A2* is associated with a decrease in plasma concentrations of enalaprilat in patients with arterial hypertension treated with enalapril, which indicates a reduced function of CES1. We hypothesize that this is due to allele C of rs2244613 being in linkage disequilibrium with *CES1A2* or possibly another genetic variant.

Summarizing our results, we can conclude that CES1 activity is reduced against enalapril in carriers of the rs2244613 allele C, in patients with haplotype containing *CES1A2*, and in *CES1A1c* homozygotes. The rs71647871 genotype showed no effect.

*CES1* is a promising pharmacogen, and its role in personalized medicine is likely to grow. Many drugs metabolized by CES1 are widely used in clinical practice, and their action is significantly influenced by genetic polymorphisms. In addition, prodrugs are increasingly being developed that are often activated by CES1. Furthermore, the role of this enzyme in lipid metabolism suggests that CES1 is a potential drug target for the treatment of metabolic diseases. Analysis of the influence of genetic factors on CES1 activity will help expand the possibilities of personalized medicine for the individualization of treatment with the many drugs that are substrates of this enzyme.

## Figures and Tables

**Table 1:** Diplotype frequencies in the studied patients.

Diplotype	n, %	HWE χ2 *p*-Value
*CES1P1/CES1P1*	193 (67.5%)	0.12
*CES1P1/CES1A2*	79 (27.6%)	
*CES1A2/CES1A2*	14 (4.9%)	

HWE—Hardy–Weinberg equilibrium.

**Table 2:** Haplotypes of *CES1* based on the rs2244613 genotype and presence of *CES1P1* or *CES1A2*.

*CES1P1* or *CES1A2*	rs2244613	Frequency, %
*CES1P1*	A	77.27
*CES1A2*	C	18.7
*CES1P1*	C	3.85
*CES1A2*	A	0.18

**Table 3:** Linear regression analysis of peak enalaprilat concentration.

	Coefficient	Standard Error	t	*p*
(Intercept)	1.412	0.29	4.875	<0.001
Age	0.013	0.004	3.467	<0.001
Sex_male	−0.192	0.08	−2.405	0.017
Single dose, ln	0.727	0.064	11.341	<0.001
*wt/CES1A1c*	0.008	0.083	0.094	0.925
*CES1A1c/CES1A1c*	−0.541	0.261	−2.073	0.039
*CES1A2*_1 copy	−0.25	0.083	−2.991	0.003
*CES1A2*_2 copies	−0.389	0.175	−2.225	0.027
Adjusted R^2^ = 0.37, *p* < 0.001				

**Table 4:** Linear regression analysis of trough enalaprilat concentration.

	Coefficient	Standard Error	t	*p*
(Intercept)	0.358	0.302	1.184	0.238
Age	0.023	0.004	5.947	<0.001
Single dose, ln	0.554	0.069	8.035	<0.001
*wt/CES1A1c*	−0.109	0.088	−1.233	0.219
*CES1A1c/CES1A1c*	−0.692	0.294	−2.352	0.019
*CES1A2*_1 copy	−0.098	0.089	−1.097	0.274
*CES1A2*_2 copies	−0.555	0.187	−2.972	0.003
Adjusted R^2^ = 0.294, *p* < 0.001				

**Table 5:** Comparison of linear regression models for peak and trough enalaprilat concentrations, using *CES1A2* or rs2244613 as predictors.

Model	R^2^	Adjusted R^2^	R^2^ Change	*p*
Peak enalaprilat concentration				
H0 (age, sex, dose(ln), *CES1Ac*)	0.359	0.348	0.359	<0.001
Adding *CES1A2* to H0	0.386	0.37	0.027	0.003
Adding *CES1* rs2244613 to H0	0.383	0.368	0.024	0.004
Trough enalaprilat concentration				
H0 (age, dose(ln), *CES1Ac*)	0.285	0.274	0.285	<0.001
Adding *CES1A2* to H0	0.31	0.294	0.025	0.01
Adding *CES1* rs2244613 to H0	0.312	0.296	0.026	0.008

## Data Availability

The data presented in this study are available on request from the corresponding author.

## Supplementary Materials

The following supporting information can be downloaded at: https://www.mdpi.com/article/10.3390/genes13122225/s1, Figure S1. Linkage disequilibrium analysis of studied variants in the *CES1* gene.
