# Supplementary figures and images for: The Influence of Structural Variants of the CES1 Gene on the Pharmacokinetics of Enalapril, Presumably Due to Linkage Disequilibrium with the Intronic rs2244613
Source: Genes (Basel). 2022 Nov 27;13(12):2225. doi: 10.3390/genes13122225 (PMC9778508; doi:10.3390/genes13122225)

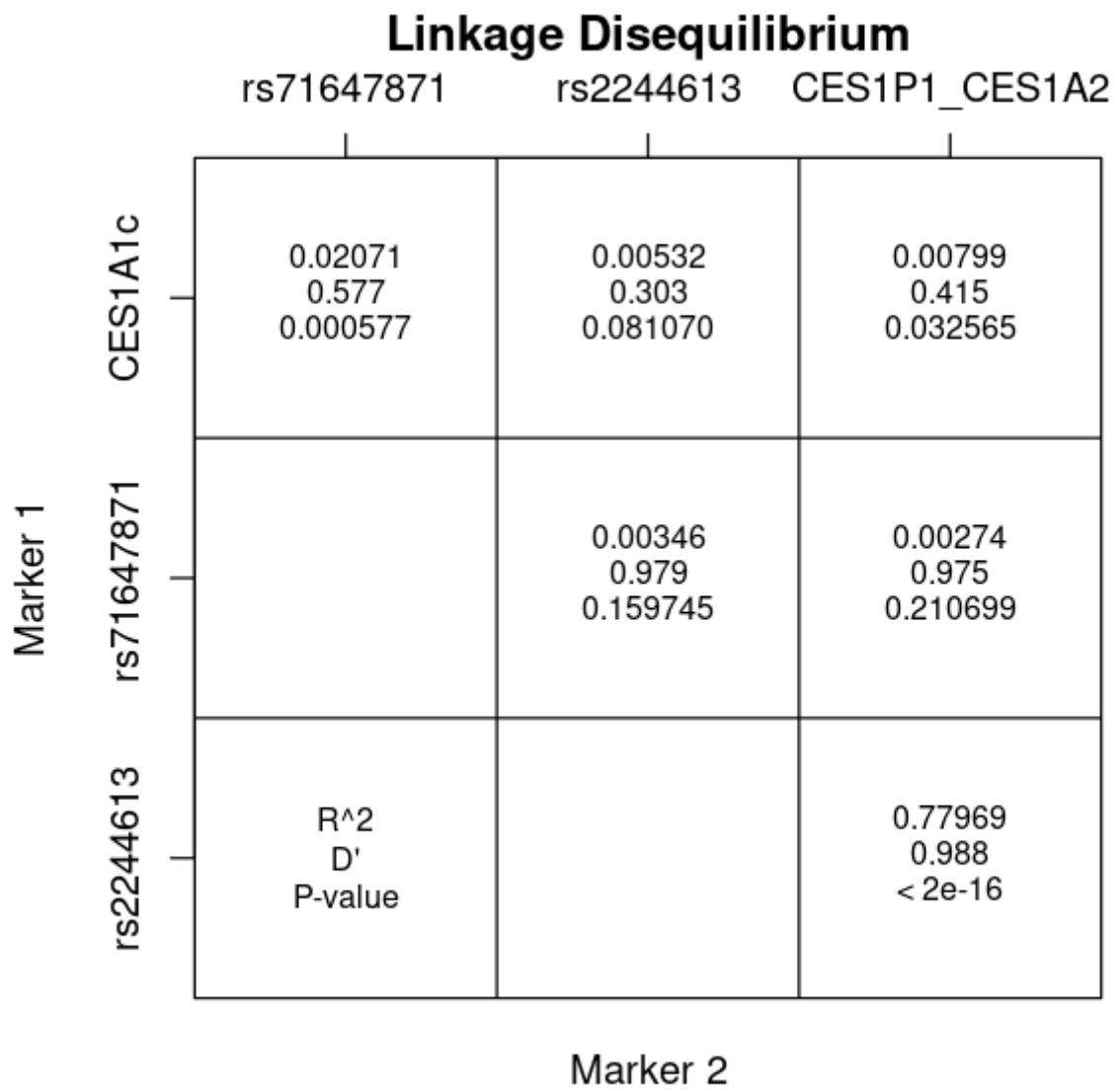

**Figure S1.** Linkage disequilibrium analysis of studied variants in the *CES1* gene.

Supplement: Supplementary file 1 [file genes-13-02225-s001.zip › genes-1996343-supplementary.pdf]
